# Epigallocatechin-3-gallate suppresses the expression of HSP70 and HSP90 and exhibits anti-tumor activity *in vitro *and *in vivo*

**DOI:** 10.1186/1471-2407-10-276

**Published:** 2010-06-10

**Authors:** Phan LCHB Tran, Soo-A Kim, Hong Seok Choi, Jung-Hoon Yoon, Sang-Gun Ahn

**Affiliations:** 1 Department of Pathology, Chosun University, College of Dentistry, Gwangju 501-759, Republic of Korea; 2 Department of Biochemistry, Dongguk University, College of Oriental Medicine, Gyeongju 780-714, Republic of Korea; 3 Chosun University, College of Pharmacy, Gwangju 501-759, Republic of Korea

## Abstract

**Background:**

Epigallocatechin-3-gallate (EGCG), one of the major catechins in green tea, is a potential chemopreventive agent for various cancers. The aim of this study was to examine the effect of EGCG on the expression of heat shock proteins (HSPs) and tumor suppression.

**Methods:**

Cell colony formation was evaluated by a soft agar assay. Transcriptional activity of HSP70 and HSP90 was determined by luciferase reporter assay. An EGCG-HSPs complex was prepared using EGCG attached to the cyanogen bromide (CNBr)-activated Sepharose 4B. *In vivo *effect of EGCG on tumor growth was examined in a xenograft model.

**Results:**

Treatment with EGCG decreased cell proliferation and colony formation of MCF-7 human breast cancer cells. EGCG specifically inhibited the expression of HSP70 and HSP90 by inhibiting the promoter activity of HSP70 and HSP90. Pretreatment with EGCG increased the stress sensitivity of MCF-7 cells upon heat shock (44°C for 1 h) or oxidative stress (H_2_O_2_, 500 μM for 24 h). Moreover, treatment with EGCG (10 mg/kg) in a xenograft model resulted in delayed tumor incidence and reduced tumor size, as well as the inhibition of HSP70 and HSP90 expression.

**Conclusions:**

Overall, these findings demonstrate that HSP70 and HSP90 are potent molecular targets of EGCG and suggest EGCG as a drug candidate for the treatment of human cancer.

## Background

Epigallocatechin-3-gallate (EGCG), one of the most abundant polyphenols in green tea, inhibits cell proliferation and induces apoptosis in a variety of human cancer cells [1-3]. Previous studies have suggested that EGCG produces anti-cancer effect by modulating the activity of mitogen-activated protein kinases (MAPKs), IGF/IGF-1 receptor, Akt, NF-κB, and CDKs [4-7]. EGCG also has other effects such as the inhibition of growth factor receptor, proteasome inhibition, mitochondrial depolarization, and the inhibition of fatty acid synthase [8-11]. Some studies have demonstrated that EGCG can inhibit the transcriptional activity of aryl hydrocarbon receptor (AhR) through the mechanism that involves direct binding of EGCG to the C-terminal region of heat shock protein 90 (HSP90) [12]. EGCG also modifies the association of HSP90 with several co-chaperones such as p23 and Hsc70, and functionally inhibits Glucose-regulated protein 78 (Grp78), a member of HSP70 family, by competing with ATP for binding to Grp78's active site [13,14]. Recent studies have implicated EGCG in the inhibition of estrogen receptor alpha (ERα), multidrug resistance protein 1 (MDR1), and telomerase in human breast cancer cells and drug-resistant breast cancer cells, leading to the suppression of cell viability and induction of apoptosis [15-17]. However, the mechanisms and signaling pathways underlying the potential anti-cancer effects of EGCG in breast cancer cells remain unclear.

Stress response upon variety of physiological and environmental stimulus including hypoxia, radiotherapy, and chemotherapy is important for cell survival [18]. HSPs are a class of stress-inducible proteins that play critical roles in stress response. HSPs function as molecular chaperone and protect cells against proteotoxic damages [19,20]. The overproduction of HSPs results in the increased incidence of cell transformation and is clinically correlated with poor prognosis and resistance to apoptosis in a wide range of human cancers [21-24]. Therefore, understanding the regulatory mechanism of HSPs and its response against anti-cancer therapies is important for the development of anti-cancer strategy.

The aim of this study was to evaluate the effects of EGCG on the expression and activity of HSPs. Here we reports that the anti-tumor activity of EGCG is mediated by the targeting of HSP70 and HSP90 *in vitro *and *in vivo *and suggests the potential value of EGCG as a therapeutic agent for cancer treatment.

## Methods

### Cell culture

The human breast cancer MCF-7 cells, mouse breast cancer 4T1 cells, and mouse colon carcinoma CT26 cells were cultured at 37°C with 5% CO_2 _in DMEM supplemented with 10% fetal bovine serum, 100 units/ml of penicillin, and 100 μg/ml of streptomycin.

### Cell proliferation assay

A total of 3 × 10^5 ^MCF-7 cells were cultured in the presence or absence of EGCG (Sigma Chemical Co., Saint Louis, MO) for 24 h. After the respective medium was removed, the cells were incubated with MTT (3-[4,5-dimethylthiazol-2-yl]-2,5-diphenyl tetrazolium bromide) solution (5 mg/ml in phosphate-buffered saline, PBS) for 3 h, and the absorbance was measured using an auto ELISA plate reader at 570 nm.

### Flow cytometric analysis

Cells were harvested and fixed with 70% ethanol for 1 h at 4°C. After washing with cold PBS, cells were incubated with DNase-free RNase and propidium iodide at 37°C for 30 min. Cells were then analyzed by flow cytometry using Cell Lab Quanta™ SC (Beckman Coulter Inc, Fullerton, CA).

### Cell colony formation assay

The inhibition of the colony formation of MCF-7 cells following treatment with EGCG was measured by soft agar assay. Briefly, cells (8 × 10^3 ^cells/ml) were treated with various concentrations of EGCG in 0.3% Basal Medium Eagle (BME) agar containing 10% FBS, 2 mM L-glutamine, and 25 μg/ml gentamicin. The cultures were maintained at 37°C with 5% CO_2 _atmosphere for 10 days. Cell colonies were scored using conventional microscope.

### Western blotting

Cells were treated with EGCG for 24 h. Cells were then washed with PBS and harvested in lysis buffer. Samples containing an equal amount of proteins were loaded into each lane of a SDS-polyacrylamide gel for electrophoresis and subsequently transferred onto a polyvinylidene difluoride membrane. After blocking, the membranes were incubated with antibodies against HSP27, HSP40, HSP60, HSP70, HSP90, HSP110, HSF1, HSF2, and β-actin (Santa Cruz Biotechnology, Santa Cruz, CA).

### Luciferase reporter assay

The promoter region of HSP70 was amplified by PCR according to the published sequence [25] and inserted into pGL3-basic luciferase reporter vector (Promega, Madison, WI). Reporter vector containing HSP90 promoter (pXP2-HSP90) was kindly provided by Wu K.J. (NYMU, Taipei). Plasmids were transfected into MCF-7 cells using FuGene 6 reagent (Roche Molecular Biochemicals, Indianapolis, IN) according to the manufacturer's instructions. After 24 h, cells were heat shocked at 42°C for 1 h. Finally, the cells were treated with or without EGCG for 24 h and lysed using lysis buffer. Luciferase activity was measured using a TriStar LB 941 multimode microplate reader (Berthold Technologies, Germany).

### EGCG-Sepharose 4B generation and *in vitro *EGCG pull-down assay

EGCG was conjugated with cyanogen bromide (CNBr)-activated Sepharose 4B (Sigma Chemical Co.). Briefly, EGCG (2.5 mg) was dissolved in 500 μl of coupling buffer (0.1 M NaHCO_3 _and 0.5 M NaCl, pH 6.0). The CNBr-activated Sepharose 4B was swelled and washed in 1 mM HCl on a sintered glass filter, then washed with the coupling buffer. CNBr-activated Sepharose 4B beads were added to the EGCG-containing coupling buffer and incubated at 4°C for 24 h. The EGCG-conjugated Sepharose 4B was washed with three cycles of alternating pH wash buffers (buffer 1, 0.1 M acetate and 0.5 M NaCl, pH 4.0; buffer 2, 0.1 M Tris-HCl and 0.5 M NaCl, pH 8.0). EGCG-conjugated beads were then equilibrated with binding buffer (0.05 M Tris-HCl and 0.15 M NaCl, pH 7.5). The control unconjugated CNBr-activated Sepharose 4B beads were prepared as described above in the absence of EGCG. The cell lysate was mixed with EGCG-conjugated Sepharose 4B in the absence or presence of ATP at 4°C for 3 h. The beads were then washed three times with binding buffer. The bound proteins were eluted with SDS loading buffer. The proteins were then resolved by SDS-PAGE followed by immunoblotting with antibodies against HSP70 and HSP90 (Santa Cruz Biotechnology).

### Xenograft model and EGCG treatment

Six-week-old male BALB/c mice were obtained from Samtako (Korea). CT26 cells (5 × 10^6 ^cells/200 μl) were injected subcutaneously and permitted to grow until palpable (4 days). When tumors reach the size of 50~150 mm^3^, the mice were randomly grouped and treated with EGCG (10 mg/kg) by daily intraperitoneal injection for 7 days. Control animals received an injection of PBS in volumes equivalent to those used for injection of EGCG (*n *= 5 for each group). The mice were observed daily for tumor growth. The tumor volume was calculated by the formula: *V *= (*ab*^2^)/2, in which '*a*' is the longest diameter and '*b*' is the shortest diameter of the tumor.

### Immunohistochemistry assay

The excised tumors were fixed in 10% formalin and embedded in paraffin. For immunohistochemical staining, avidin-biotin complex method was performed using anti-HSP70, anti-HSP90, and anti-PCNA antibodies (Santa Cruz Biotechnology). The immune reactions were visualized by immersing the sectioned tissues in 3,3'-diaminobenzidine tetrahydrochloride. Counterstaining was performed with hematoxylin.

### Statistical analysis

All statistical analyses were carried out using Excel software. The significance of the differences was determined using an independent-samples *t*-test. A *p*-value < 0.05 was regarded as statistically significant.

## Results

### EGCG induces the G2/M phase cell cycle arrest in MCF-7 cells

**Figure 1:**
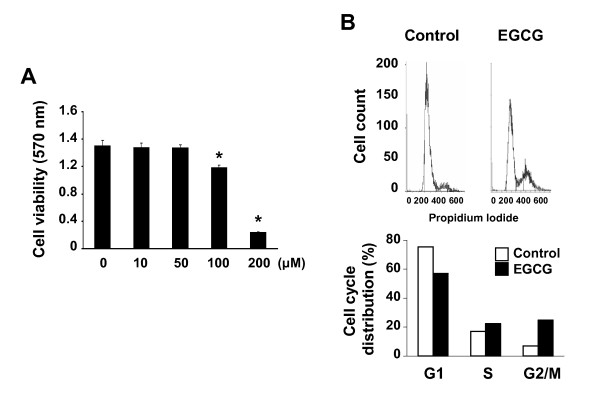
**EGCG inhibits cell proliferation and induces the G2/M phase cell cycle arrest in MCF-7 cells**. (**A**) The cells were treated with increasing concentrations of EGCG and cell viability was assessed by MTT assay. *Asterisk*, *P *< 0.05, significantly different from control. (**B**) To determine the effect of EGCG on cell cycle progression, cells were treated with 100 μM of EGCG for 24 h. Cell cycle distribution was monitored by flow cytometry.

The effect of EGCG on MCF-7 human breast cancer cells was examined by increasing concentrations of EGCG. As shown in Figure 1A, treatment with EGCG (10 ~ 200 μM) inhibited the growth of MCF-7 cells in a dose-dependent manner with an IC_50 _of 150 μM. Extensive inhibition of cell growth was observed in the cells treated with high concentration (200 μM) of EGCG. Cell cycle distribution analysis of MCF-7 cells treated with EGCG (100 μM) showed that the cells were mainly arrested at the G2/M phase (Figure 1B).

### EGCG inhibits cell colony formation of MCF-7 cells

**Figure 2:**
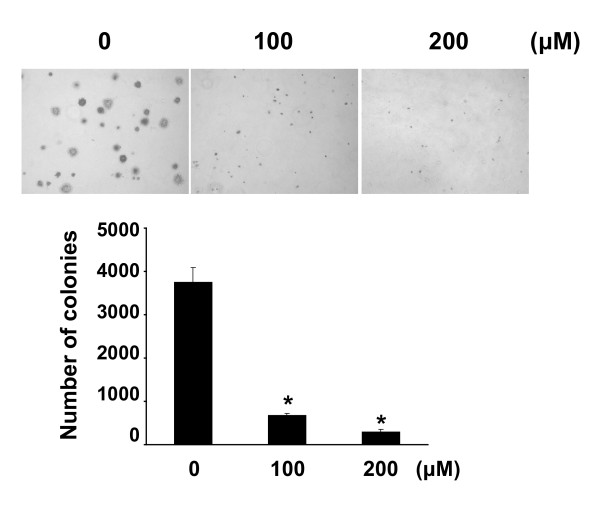
**EGCG inhibits colony formation of MCF-7 cells on soft agar**. Cell suspensions were mixed with 0.3% agar in the culture medium with EGCG. After 10 days of incubation at 37°C in a 5% CO_2 _incubator, colonies were counted. Representative phase contrast images are shown. The data are represented of the average number of colonies per plate as determined from three separate experiments. *Asterisk*, *P *< 0.001, significantly different from control.

MCF-7 cells were plated on a soft agar matrix, treated with EGCG, and incubated at 37°C in a 5% CO_2 _incubator. After 10 days, the number of colonies was counted. As shown in Figure 2, EGCG inhibited colony formation in a dose-dependent manner, suggesting that EGCG is a critical inhibitor of MCF-7 cell proliferation.

### EGCG inhibits the expression of HSP70 and HSP90 in MCF-7 cells

**Figure 3:**
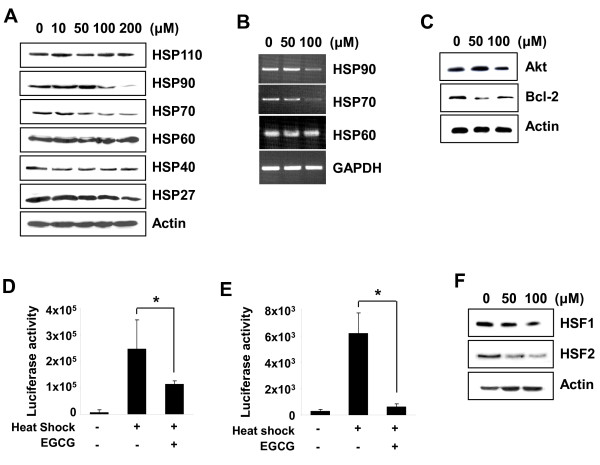
**FEGCG suppresses the expression of HSP70 and HSP90 in MCF-7 cells**. (**A**) The cells were treated with EGCG for 24 h and the expression levels of heat shock proteins were determined by Western blot analysis. (**B**) The levels of HSP90, HSP70, and HSP60 mRNA were detected by RT-PCR analysis. GAPDH was used as an internal control. (**C**) The cells were harvested 24 h after EGCG treatment. Cell lysates were subjected to Western blot analysis for Akt and Bcl-2. (**D**), (**E**) MCF-7 cells were transfected with pGL3-HSP70 (D) or pXP2-HSP90 (E) reporter vector. After 24 h, cells were heat shocked at 42°C for 1 h followed by recovery at 37°C for 24 h with EGCG (100 μM). Cells were harvested and the cell extracts were subjected to luciferase assay. The results were represented as the mean ± SD of three independent experiments. *Asterisk*, *P *< 0.05, significantly different from control. (**F**) The cells were treated with indicated concentrations of EGCG for 24 h. The expression levels of HSF1 and HSF2 were monitored by Western blot analysis.

Several reports have revealed that HSPs are important mediators of chemotherapy resistance. Therefore, we investigated the effect of EGCG on the expression of various HSPs. MCF-7 cells were incubated with increasing concentrations of EGCG (10 ~ 200 μM) for 24 h. As shown in Figure 3A and 3B, the levels of protein and mRNA of HSP70 and HSP90 were decreased by 100 μM of EGCG, while the other HSPs (HSP110, HSP60, HSP40, and HSP27) were unaffected. In addition, EGCG inhibited the expression of HSP90-regulated Akt and Bcl-2 in MCF-7 cells (Figure 3C).

To examine the effect of EGCG on the promoter activity of HSP70 and HSP90, we performed the luciferase reporter assay. Consistent with the previous studies, heat shock (42°C for 1 h) induced transcriptional activity of HSP70 and HSP90 (Figure 3D and 3E). However, heat shock-induced promoter activity was suppressed by EGCG.

To understand the molecular changes associated with HSP70 and HSP90 inhibition, we examined the protein levels of HSP transcription factors. As shown in Figure 3F, EGCG significantly decreased the levels of heat shock transcription factor 1 (HSF1) and HSF2 in a dose-dependent manner. These data suggest that EGCG suppresses the expression of HSP70 and HSP90 by inhibiting the expression of their transcription factors, HSF1 and HSF2.

### EGCG competes with ATP for binding to the ATPase domain of HSP70 and HSP90

**Figure 4:**
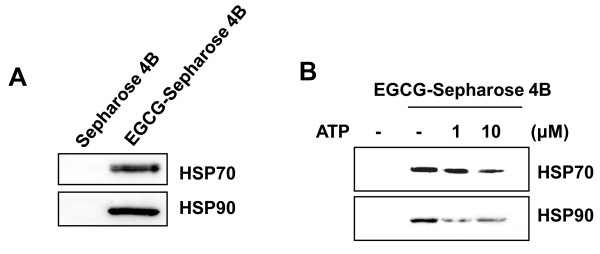
**EGCG binds to the ATPase domain of HSP70 and HSP90**. (**A**) Whole cell lysates were incubated with EGCG-conjugated Sepharose 4B. After precipitation, the levels of bound HSP70 or HSP90 were monitored by Western blot analysis. (**B**) Whole cell lysates were incubated with EGCG-Sepharose 4B in the absence or presence of ATP. The levels of bound HSP70 or HSP90 in precipitates were monitored by Western blot analysis.

It was previously shown that EGCG antagonizes the function of the HSP70 family protein, Grp78, by directly competing with ATP for binding to the ATPase domain of Grp78 [14]. To investigate this possibility, we assessed the interaction between the HSPs and EGCG using EGCG-conjugated Sepharose 4B beads. EGCG-conjugated Sepharose pull-down assay confirmed that both HSP70 and HSP90 interact efficiently with EGCG (Figure 4A). Then, we examined the competition between EGCG and ATP for the ATPase binding pockets of HSP70 and HSP90. As shown in Figure 4B, the binding of EGCG with HSP70 or HSP90 was decreased with increasing amount of ATP. These data demonstrate that EGCG competes with ATP for binding to the ATPase domain of HSP70 and HSP90.

### Stress sensitivity is increased in EGCG-treated MCF-7 cells

**Figure 5:**
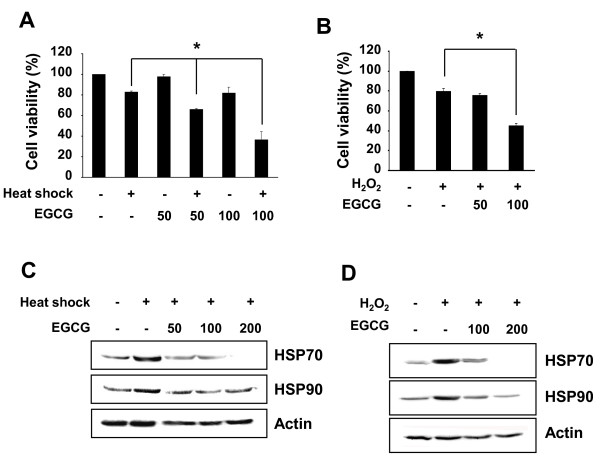
**EGCG increases the stress sensitivity of MCF-7 cells**. The cells were treated with EGCG for 24 h and then heat shocked (44°C for 1 h) or treated with H_2_O_2 _(500 μM, 37°C for 24 h). (**A**), (**B**) Cell viability was measured by MTT assay. *Asterisk*, *P *< 0.005, significantly different from the cells treated with heat shock or H_2_O_2 _alone. (**C**), (**D**) The levels of HSP70 and HSP90 were monitored by Western blot analysis.

Next, we examined whether EGCG has effect on the cell viability upon heat shock or H_2_O_2 _treatment. MCF-7 cells were pretreated with EGCG for 24 h and the cells were heat shocked (44°C, 1 h) or treated with H_2_O_2 _(500 μM, 24 h). Upon stress, cell viability was decreased approximately 20% in MCF-7 cells (Figure 5A and 5B). Interestingly, pretreatment of cells with EGCG strongly reduced the cell viability after heat shock or H_2_O_2 _treatment compared with single treatment with EGCG or stress (Figure 5A and 5B). The inhibition of cell growth was increased depending on the EGCG concentration. In addition, heat shock- and H_2_O_2_-induced HSP70 and HSP90 expression were significantly inhibited in the cells pretreated with EGCG (Figure 5C and 5D). These results indicate that EGCG increases the stress sensitivity of MCF-7 cells through the suppression of HSP70 and HSP90 expression.

### EGCG suppresses tumor growth in xenograft model

**Figure 6:**
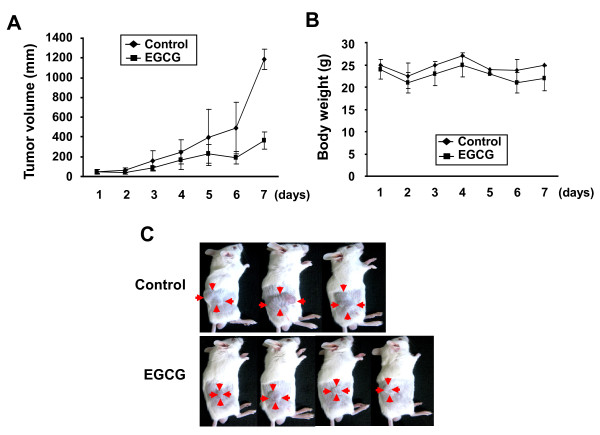
**EGCG represses the growth of tumor in mice**. BALB/c mice were subcutaneously injected with 5 × 10^6 ^colon carcinoma CT26 cells into the right flank. After 4 days, the mice were given daily dose of PBS or EGCG (10 mg/kg) through intraperitoneal injection for 7 days as described in "Methods" section. (**A**), (**B**) Serial tumor volumes and body weights were measured everyday. Values represent mean ± SD. (**C**) Representative images of xenograft tumors.

The anti-tumor efficacy of EGCG was examined on a mouse xenograft model using CT26 colon cancer cells. CT26 cells were injected subcutaneously in BALB/c mice. After the palpable tumors were appeared (4 days after injection), EGCG (10 mg/kg) was injected intraperitoneally every day for 7 days. As shown in Figure 6A, the administration of EGCG caused a 70% decrease in tumor volume compared with PBS-treated control mice. The toxicity of EGCG was assessed by mouse survival and careful monitoring of body weight. The EGCG treatment did not alter body weight compared with PBS-treated control mice (Figure 6B).

**Figure 7:**
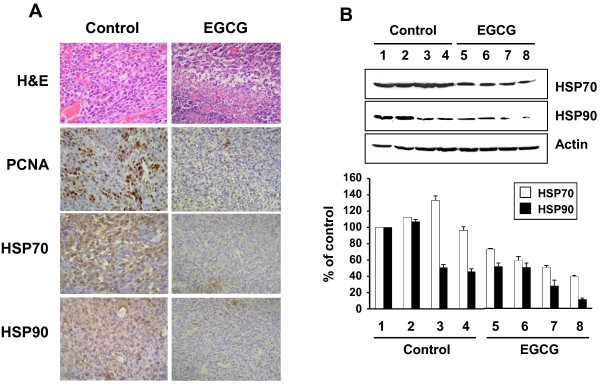
**Histological and immunohistochemical appearance of tumors from CT26 cell-injected mice**. Mice were subcutaneously injected with CT26 cells into the right flank. Four days later, mice were treated with either PBS (control) or EGCG for 7 days. (**A**) H & E staining and immunohistochemical staining of PCNA, HSP70, and HSP90 for tumor sections. (**B**) The levels of HSP70 and HSP90 in the lysates of tumor tissues were analyzed by Western blot analysis. Western blot data were quantified by densitometry. The data were expressed as the mean ± SD of three individual experiments.

To examine whether EGCG is able to inhibit the expression of HSP70 and HSP90 *in vivo*, the tumor tissues were excised. Immunohistochemistry assay showed that the levels of HSP70 and HSP90 were decreased in EGCG-treated mice compared with control (Figure 7A). The expression of PCNA, a proliferation biomarker, also significantly decreased (approximately 50%). Western blot analysis showed that the expression levels of HSP70 and HSP90 were decreased in EGCG-treated mice compared with control (Figure 7B).

## Discussion

EGCG is an anti-oxidant that plays an important role in preventing cancer and cardiovascular disease [26]. Although many different molecules and signaling pathways have been demonstrated in various cell lines, the exact action mechanism of EGCG is unknown.

Ciocca *et al*. reported that the expression of three main HSPs, HSP27, HSP70, and HSP90, are elevated in breast cancer [21]. It is known that the levels of the HSPs are elevated in many cancers, and elevated HSP expression provides cellular resistance to anti-cancer therapies [21]. Some studies have shown that EGCG inhibits tumor growth by suppressing the HSP27, HSP70, HSP90, and/or HSP90 client proteins [13,27,28]. Grp78, a member of HSP70 family, is one of the target proteins of EGCG. Grp78 plays critical role as a molecular chaperone and promotes tumor cell proliferation, survival, and metastasis [14]. Therefore, HSPs could be a possible candidate to improve the efficacy of anti-cancer therapy.

Our data demonstrated that EGCG has an anti-proliferative effect on MCF-7 cells. In addition, EGCG specifically reduced the expression of HSP70 and HSP90 without affecting the levels of other HSPs. We also observed that EGCG inhibits the expression of HSP90 client protein, Akt. Previously, it was reported that HSP90 directly interacts with Akt and this process is essential for the stability and function of Akt [29]. Although we did not directly observe the inhibition of Akt signaling by HSP90 inhibition, EGCG-induced cell cycle arrest may associate with the inhibition of HSP90/Akt signaling. Additionally, we observed that EGCG binds with HSP70 and HSP90 by using an EGCG-Sepharose 4B pull-down assay. We also found clear evidence that EGCG competes with ATP for binding to the ATP binding pocket of HSP70 and HSP90 in human breast cancer cells. Although Li *et al*. reported that EGCG has no effect on ATP binding to HSP90 [13], several studies have reported that EGCG interacts with the ATPase domain of HSP90 and Grp78 and inhibits their functions by competing with ATP for binding to the ATP binding domain in hepatoma and pancreatic cancer cells [12,14]. Collectively, these results suggest that EGCG is likely to affect signaling pathways of cell growth and survival by inhibiting the expression of HSP70 and HSP90 and/or by binding to the HSP70 and HSP90.

Here we demonstrated, for the first time, that EGCG suppresses the expression of HSF1 and HSF2. A luciferase reporter assay showed that EGCG inhibits heat shock- or oxidative stress-induced promoter activity of HSP70 and HSP90. These results demonstrate that EGCG can inhibit the upstream regulator of HSP70 and HSP90. Previous studies suggested that the level of HSF1 become elevated in a number of cancer cells, and the elevated HSF1 is implicated in tumorigenesis through the expression of the HSP70 family proteins and the inhibition of apoptotic pathways [30]. Induction of HSPs by HSF1 is essential for the growth of many tumor cells. Furthermore, inhibition of HSF1 leads to the induction of cell death and tumor regression [31-33]. Therefore, HSF1 is an attractive target for cancer treatment. Unlike HSF1, the role of HSF2 in tumor progression is unclear. Recent studies have shown that HSF1 can physically associate with HSF2 and their interaction is enhanced by heat shock [34,35]. Therefore, the interplay between HSF1 and HSF2 may regulate their function and play an important role on tumor progression by activating target genes.

It is widely accepted that HSPs play an important role in cell protection during cellular stress [18,36]. In this study, we examined the effect of EGCG on heat shock- or oxidative stress (H_2_O_2_)-treated MCF-7 cells. Heat shock and H_2_O_2 _inhibited cell viability approximately 20% and induced the expression of HSP70 and HSP90. Pretreatment with EGCG further suppressed cell viability after heat shock or H_2_O_2 _treatment. And the stress-induced expression of HSP70 and HSP90 was suppressed by EGCG pretreatment in a dose-dependent manner. These results suggest that EGCG enhances stress sensitivity of cells by suppressing the expression of HSP70 and HSP90.

Several studies have shown that the anti-tumor activity of EGCG in xenograft model [37,38]. Here we showed that EGCG inhibits tumor formation in CT26 cell inoculated BALB/c mice. Immunohistochemical staining showed decreased levels of PCNA, HSP70, and HSP90 in the tumors from EGCG treated mice. We also examined the effect of EGCG in xenograft model using 4T1 murine breast cancer cells. In our study, CT26 cells were more sensitive to EGCG than 4T1 cells *in vivo *(data not shown). Because different types of tumor cells have different genetic alterations and characteristics, the molecular targets of EGCG may be different in different cell lines. The role of HSPs in tumorigenesis is supported by the experimental data showing high expression levels of HSPs are required for the growth of tumor xenografts. Furthermore, high level of HSPs correlates with poor therapeutic outcome in human breast cancer suggesting the inhibition of HSPs can be therapeutically useful [39,40].

In this study, we demonstrate that the anti-tumor activity of EGCG is mediated by the inhibition of HSP70 and HSP90 and suggests EGCG as a potent candidate for the anti-tumor agent.

## Conclusion

In this study, we demonstrated that the treatment of EGCG decreased cell proliferation and colony formation of MCF-7 human breast cancer cells. In addition, EGCG specifically inhibited the expression of HSP70 and HSP90 by inhibiting their promoter activity. Pretreatment with EGCG increased the stress sensitivity of MCF-7 cells upon heat shock or oxidative stress. Moreover, treatment with EGCG in a xenograft model resulted in delayed tumor incidence and reduced tumor size, as well as the inhibition of HSP70 and HSP90 expression. These results suggest that HSP70 and HSP90 could be a potential molecular target of EGCG.

## Abbreviations

EGCG: Epigallocatechin-3-gallate; HSP: heat shock protein; HSF: heat shock transcription factor; MTT: 3-[4,5-dimethylthiazol-2-yl]-2,5-diphenyl tetrazolium bromide; CNBr: cyanogen bromide; PCNA: proliferating cell nuclear antigen.

## Competing interests

The authors declare that they have no competing interests.

## Authors' contributions

PLCHBT, and HSD carried out the experiments described in the study. JHY, SAK, and SGA conceived and reviewed the study. All authors read and approved the final manuscript.

## Pre-publication history

The pre-publication history for this paper can be accessed here:

http://www.biomedcentral.com/1471-2407/10/276/prepub
